# Aesthetic Improvements Over Time: Long‐Term Efficacy and Additional Outcomes of IncobotulinumtoxinA in the Simultaneous Treatment of Upper Facial Lines

**DOI:** 10.1111/jocd.70460

**Published:** 2025-09-18

**Authors:** Tatjana Pavicic, Cheryl Burgess, Sabrina Fabi, Mark S. Nestor, Eva Kristina Bee, Matthias Imhof, Hanna Dersch, Vladimir Sudimac

**Affiliations:** ^1^ Private Practice for Dermatology & Aesthetics Dr. Tatjana Pavicic Munich Germany; ^2^ Center for Dermatology and Dermatologic Surgery Washington DC USA; ^3^ Cosmetic Laser Dermatology San Diego California USA; ^4^ Center for Clinical and Cosmetic Research Aventura Florida USA; ^5^ Department of Dermatology and Cutaneous Surgery, Division of Plastic Surgery, Department of Surgery, Miller School of Medicine University of Miami Miami Florida USA; ^6^ Practice for Aesthetic Dermatology Drensteinfurt Germany; ^7^ Hautmedizin Bad Soden Aesthetic Dermatology Bad Soden Germany; ^8^ Merz Aesthetics GmbH Frankfurt Germany

**Keywords:** aesthetics, BoNT‐A, forehead, incobotulinumtoxinA, upper facial lines, Xeomin

## Abstract

**Background/Objectives:**

Two Phase III randomized controlled trials were conducted in the US (ULTRA I) and Germany (ULTRA II) to investigate the efficacy of incobotulinumtoxinA for simultaneous treatment of upper facial lines (UFLs: glabellar frown lines [GFLs], horizontal forehead lines [HFLs] and lateral canthal lines [LCLs]).

**Patients/Methods:**

Healthy adults with moderate to severe GFLs, HFLs, and symmetrical LCLs were enrolled. Participants were randomized 2:1:1 to three groups: placebo, UFLs, and GFLs & HFLs (ULTRA I) or LCLs (ULTRA II). All participants received up to 64 units of incobotulinumtoxinA in the main period (MP) and were eligible for two additional treatment cycles in the open‐label extensions (OLEX). Endpoints included participant‐ and investigator‐assessed Global Aesthetic Improvement Scale (pGAIS and iGAIS, respectively; any improvement defined as ≥ +1 score versus baseline) and the percentage of GFL, HFL, and LCL responders, defined as ≥ 1‐grade improvement in Merz Aesthetics Scale (MAS) score versus baseline at maximum contraction.

**Results:**

For pGAIS at Day 30, the proportion of participants with any improvement was very high (> 97%) across all three treatment cycles. iGAIS results were similar and slightly higher than pGAIS results. In the MP, investigator and participant MAS assessments were similar, with > 78% of participants showing a positive treatment response for UFLs up to Day 90; responses in the placebo groups remained low at all time points (< 13%). MAS results in the OLEX were consistent with the MP.

**Conclusions:**

Additional endpoints, including pGAIS, iGAIS, and MAS scores, corroborate prior ULTRAI/II findings and demonstrate the efficacy of incobotulinumtoxinA for simultaneous treatment of UFLs.

**Trial Registration:**

ULTRA I: NCT04594213; ULTRA II: NCT04622254

## 1 Introduction

During the aging process, upper facial lines (UFLs), consisting of glabellar frown lines (GFLs), lateral canthal lines (LCLs) and horizontal forehead lines (HFLs), appear and increase in prominence [1]. For patients desiring a harmonized aesthetic effect, simultaneous treatment of the UFLs with botulinum toxins is gaining popularity due to the ability to maintain facial proportions [2, 3].

IncobotulinumtoxinA (Xeomin) is a lyophilized formulation of botulinum toxin type A (BoNT‐A), which is highly purified and does not contain complexing proteins [4, 5]. This is beneficial as complexing proteins may induce neutralizing antibodies, which can lead to secondary treatment failure [6]. Further, stability is unaffected by the absence of complexing proteins [7], with a previous study determining that prior to reconstitution, incobotulinumtoxinA remained stable up to 48 months without the need for refrigeration [5].

In 2016, incobotulinumtoxinA received approval in the European Union for the simultaneous treatment of the UFLs [8]. Approval in the US for the same indication was granted in 2024 following the positive results from two Phase III trials, which provided detailed long‐term efficacy and safety data for the simultaneous injection of incobotulinumtoxinA into UFLs; one study was conducted in the US (ULTRA I) and the other in Germany (ULTRA II) [9, 10].

The current manuscript details additional secondary efficacy endpoints from the main treatment periods of both ULTRA trials and their respective open‐label extensions (OLEX); these results were not reported in the primary publication [10].

## 2 Materials and Methods

### 2.1 Study Design

ULTRA I and ULTRA II were prospective, randomized, double‐blind, placebo‐controlled, multicenter studies conducted across 12 sites in the US (ULTRA I; NCT04594213) and 12 sites in Germany (ULTRA II; NCT04622254).

In brief, both studies included a main period (MP) with one incobotulinumtoxinA (Merz Pharmaceuticals GmbH, Frankfurt, Germany) injection cycle (120 days plus screening [Days −14 to −3]). Participants were followed for 120 days and eligibility for reinjection was confirmed before the respective OLEX. The OLEX consisted of two incobotulinumtoxinA injection cycles (120 days plus up to 30 days for eligibility reassessment in Cycle 2 or 120 days ±7 days in Cycle 3, end of treatment). In each trial's MP, participants were randomized 2:1:1 into three treatment groups. The UFLs treatment group (Group U; both trials) received a total dose of 64 units (U) of incobotulinumtoxinA as simultaneous injections in all three facial areas: 20 U in the GFLs, 20 U in the HFLs, and 24 U in the LCLs. The placebo group (Group P; both trials) received placebo in all three facial areas. The GFLs and HFLs treatment group (Group G&H, ULTRA I only) received a total dose of 40 U: 20 U each in the GFLs and HFLs and placebo in the LCLs. The LCLs treatment group (Group L, ULTRA II only) received a total dose of 24 U in the LCLs: 12 U each side and placebo in the GFLs and HFLs.

The protocols were approved by the institutional review boards and/or independent ethics committees. All participants provided written informed consent and the study was conducted in compliance with Good Clinical Practice and the Declaration of Helsinki.

### 2.2 Participants

Participants were male and female, aged ≥ 18 years with HFLs, GFLs, and symmetrical LCLs of moderate (score 2) to severe (score 3) intensity at maximum contraction, as assessed by the investigator and participant, according to the validated five‐point Merz Aesthetics Scales (MAS) [11, 12] at baseline.

### 2.3 Treatment

IncobotulinumtoxinA (100 U) was reconstituted with 2.5 mL unpreserved sterile physiological (0.9%) sodium chloride solution for injection; placebo was provided in identical vials with excipients only (sucrose, human serum albumin). Both were manufactured by Merz Pharmaceuticals GmbH, Eckenheimer Landstr. 100, D‐60318 Frankfurt am Main, Germany. In the MP, 0.1 mL of incobotulinumtoxinA or placebo was administered to each injection point. In the OLEX, 0.1 mL of incobotulinumtoxinA was administered to each injection point.

### 2.4 Assessments

At the baseline visit (before injection) and at each posttreatment visit, standardized 2D participant photographs were collected at rest and at maximum contraction. Safety was assessed throughout both trials; results are reported in the primary publication [10].

### 2.5 Global Aesthetic Improvement Scale

Using baseline photographs for reference, investigators and participants assessed aesthetic improvement after treatment using the balanced 7‐point Likert Global Aesthetic Improvement Scale (GAIS; range −3 to +3, where −3: very much worse to +3: very much improved).

Improvement in UFLs appearance was assessed by the participants (pGAIS) and investigator (iGAIS) at Days 8, 30, 60, 90, and 120 in the MP and Days 8, 30, 75, and 120 in the OLEX. Any improvement was defined as a score of at least +1 versus baseline.

### 2.6 Merz Aesthetics Scale

Severity of the GFLs, HFLs, and LCLs (left and right side separately) was assessed at scheduled visits, using the investigator's and participant's live assessment, according to the validated 5‐point MAS for UFLs. On the MAS, 0 corresponds to “no lines,” 1 “mild lines,” 2 “moderate lines,” 3 “severe lines,” and 4 “very severe lines” [11, 12]. Investigators were trained and qualified on the MAS before evaluating participants.

In the MP (Cycle 1) and OLEX (Cycles 2 and 3), the MAS was used to assess GFLs, HFLs, and LCLs for each time point (Days 8, 30, 60, 90, and 120 for MP and Day 30 for both cycles in OLEX), rating condition (at maximum contraction), and rater (investigator, participant). A positive treatment response was defined as the rate (expressed as percentage) of participants with a MAS score of at least 1‐grade improvement from baseline for the respective area at each time point.

### 2.7 Statistical Analysis

Efficacy endpoints in this publication were analyzed descriptively using observed cases, as confirmatory testing or type I error adjustment was performed only for the previously published primary and key secondary endpoints [10]. Analysis of MP‐efficacy endpoints was conducted using the full analysis set (FAS), which included all randomized participants. Analyses of OLEX efficacy data included participants treated in the respective cycle (i.e., a subset of the FAS). Two‐sided 95% Wilson confidence intervals (CIs) for proportions were provided. For the MP only, unadjusted differences in proportions between Group U and Group P with 95% Mantel–Haenszel CIs for differences were calculated. For GAIS in the MP, an additional pooled group for those who received any treatment with incobotulinumtoxinA was calculated (Group U and G&H combined for ULTRA I and Group U and L combined for ULTRA II). In the OLEX periods, participants from the three MP treatment groups were pooled (Total Inco) and no differences in proportions were calculated.

## 3 Results

### 3.1 Participant Demographics

A total of 362 participants in the US were randomized in ULTRA I, and a total of 368 participants in Germany were randomized in ULTRA II. A total of 226 participants (62.4%) in ULTRA I and 313 participants (85.1%) in ULTRA II completed the trial, including the OLEX. Reasons for discontinuations were previously reported [10]. In brief, the mean participant age in ULTRA I was 47.4 years (range, 22–76 years), 85.1% were female and 27.3% had received prior BoNT‐A treatment. In ULTRA II, mean age was 45.7 years (range, 19–70 years), 82.3% were female and 39.4% had received prior BoNT‐A treatment. Detailed participant disposition, demographics, and clinical characteristics were previously reported [10].

### 3.2 GAIS

Overall, the rate of participants with any improvement (score of at least +1) in pGAIS at Day 30 was very high across all three cycles: > 97% for ULTRA I and > 98% for ULTRA II among those treated with incobotulinumtoxinA (Table 1). At MP Day 30, the rate of participants with pGAIS improvement was higher in Group U versus Group P in ULTRA I (unadjusted difference: 90.9% [95% CI: 85.3, 96.6]) and ULTRA II (96.2% [95% CI: 92.7, 99.7]).

**TABLE 1:** pGAIS, “any improvement”^a^ over three treatment cycles.

Day	ULTRA I						ULTRA II					
	Treatment	*n*/*n*‐obs	%	95% CI^b^	Unadjusted (vs. Group P)^c^		Treatment	*n*/*n*‐obs	%	95% CI^b^	Unadjusted (vs. Group P)^c^	
					Difference in proportions	95% CI					Difference in proportions	95% CI
MP Day 30	Group P^d^ (*N* = 91)	6/89	6.7	3.1, 13.9			Group P^d^ (*N* = 94)	2/92	2.2	0.6, 7.6		
MP Day 30	Group U^d^ (*N* = 179)	168/173	97.7	94.2, 99.1	90.9	85.3, 96.6	Group U^d^ (*N* = 184)	178/181	98.3	95.2, 99.4	96.2	92.7, 99.7
Cycle 2 Day 30	Total Inco^e^ (*N* = 303)	283/290	97.6	95.1, 98.8			Total Inco^e^ (*N* = 346)	344/344	100.0	98.9, 100.0		
Cycle 3 Day 30	Total Inco^e^ (*N* = 238)	218/224	97.3	94.3, 98.8			Total Inco^e^ (*N* = 318)	308/313	98.4	96.3, 99.3		

*Note:* This table presents the rate of participants with “any improvement”^a^ on the GAIS, as assessed by the participant, over the MP and OLEX cycles. In the OLEX cycles, as treatment was open‐label, participants were pooled into one treatment group, which was renamed Total Inco. Analysis is based on observed cases in the full analysis set.

Abbreviations: CI, confidence interval; Inco, incobotulinumtoxinA; MP, main period; *n*, number of responders; *N*, number of participants in respective analysis set; *n*‐obs, number of observed cases; OLEX, open‐label extension; pGAIS, participant‐assessed Global Aesthetic Improvement Scale.

^a^ Score of at least +1.

^b^ Wilson CI.

^c^ CI is based on the Mantel–Haenszel method without stratum adjustment.

^d^ Group P: placebo group, Group U: upper facial lines (a combination of glabellar frown lines and horizontal forehead lines and lateral canthal lines) treated.

^e^ Total Inco: MP treatment groups were pooled for the OLEX cycles.

**FIGURE 1:**
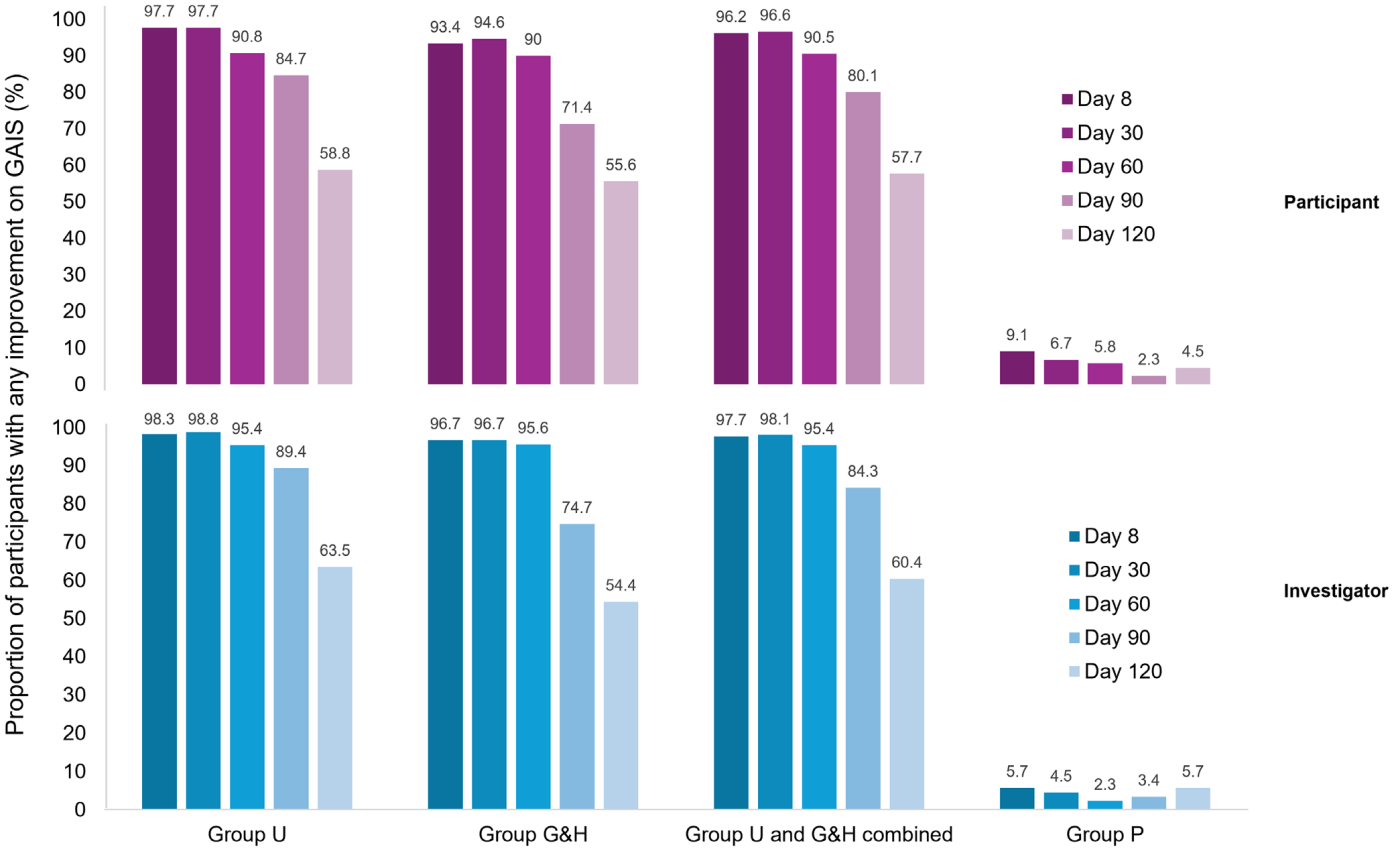
GAIS, “any improvement”^a^ over ULTRA I MP. This figure presents the rate of participants with “any improvement”^a^ on the GAIS in the MP of ULTRA I, as assessed by the participant (above, purple) and investigator (below, blue). To see the contrast with the placebo group, all participants who received treatment are presented as “Group U and G&H combined.” Analysis is based on observed cases in the full analysis set. ^a^Score of at least +1. GAIS, Global Aesthetic Improvement Scale; Group G&H, treatment of glabellar frown lines and horizontal forehead lines and placebo treatment of lateral canthal lines; Group P, placebo group; Group U, upper facial lines (a combination of glabellar frown lines and horizontal forehead lines and lateral canthal lines) treated; MP, main period; *N*, number of participants in respective analysis set.

The rate of ULTRA I participants with any pGAIS improvement during the MP is shown in Figure 1. Through Day 60, the majority of participants (≥ 90.0%) in the incobotulinumtoxinA treatment groups achieved an improvement. By Day 90, the rate of participants with any pGAIS improvement declined to 71.4% and 84.7% for Group G&H and Group U, respectively. Similarly, by Day 120, the rates declined to 55.6% and 58.8% for Group G&H and Group U, respectively. Overall, the iGAIS results were similar and slightly higher than pGAIS results (Figure 1).

**FIGURE 2:**
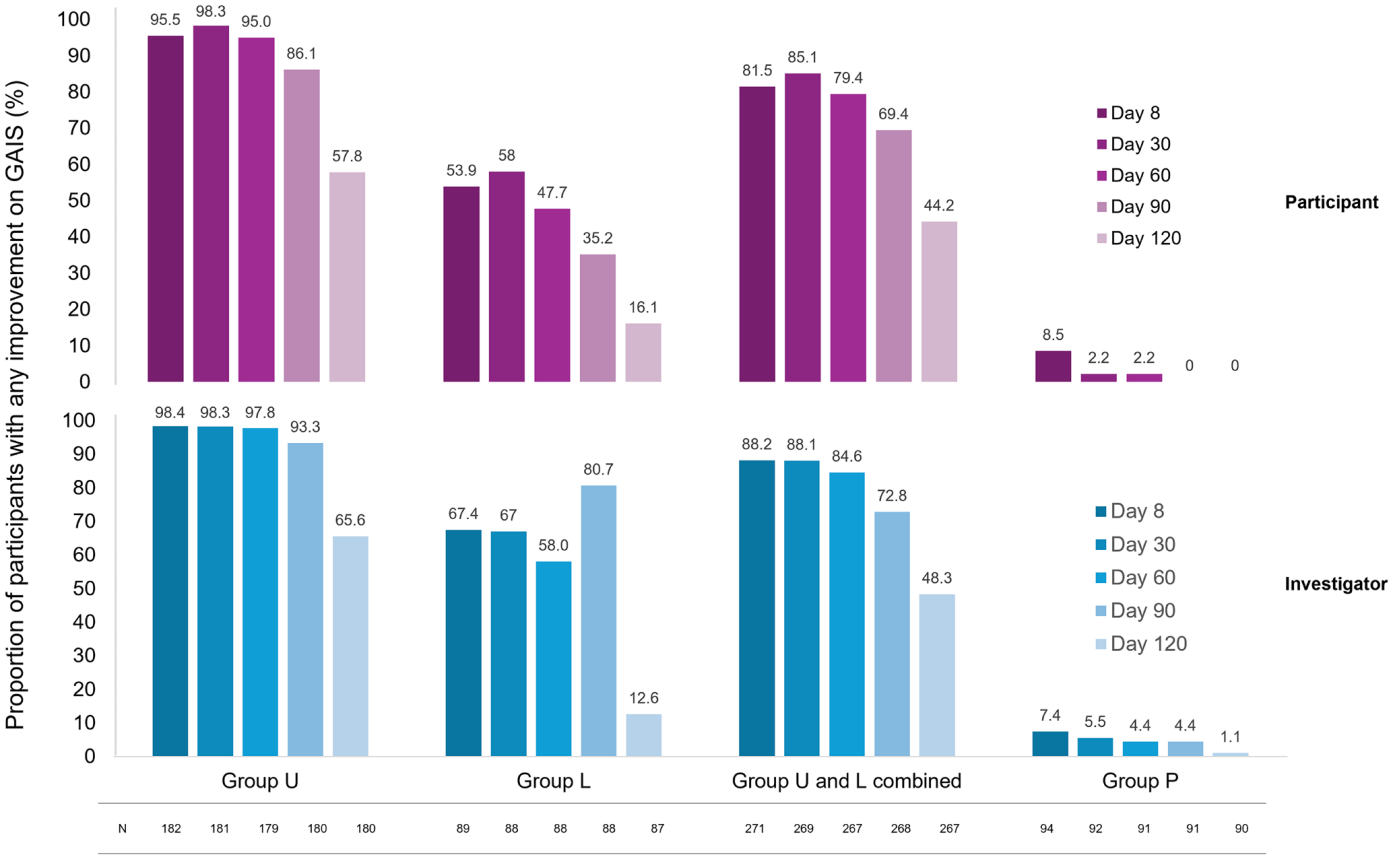
GAIS, “any improvement”^a^ over ULTRA II MP. This figure presents the rate of participants with “any improvement”^a^ on the GAIS in the MP of ULTRA II, as assessed by the participant (above, purple) and investigator (below, blue). To see the contrast with the placebo group, all participants who received treatment are presented as “Group U and L combined”. Analysis is based on observed cases in the full analysis set. ^a^Score of at least +1. GAIS, Global Aesthetic Improvement Scale; Group L, treatment of lateral canthal lines; Group P, placebo group; Group U, upper facial lines (a combination of glabellar frown lines and horizontal forehead lines and lateral canthal lines) treated; MP; main period; *N*, number of participants in respective analysis set.

In ULTRA II, the rate of participants with any improvement in pGAIS during the MP is shown in Figure 2. Through Day 60, most participants in the incobotulinumtoxinA treatment groups achieved any improvement (≥ 79% for Groups U and L combined). For Group U, the rate of participants with any pGAIS improvement was 86.1% by Day 90 and 57.8% by Day 120. For Group L, the rates were lower across all time points. Overall, the iGAIS results were similar and slightly higher than pGAIS results (Figure 2).

**FIGURE 3:**
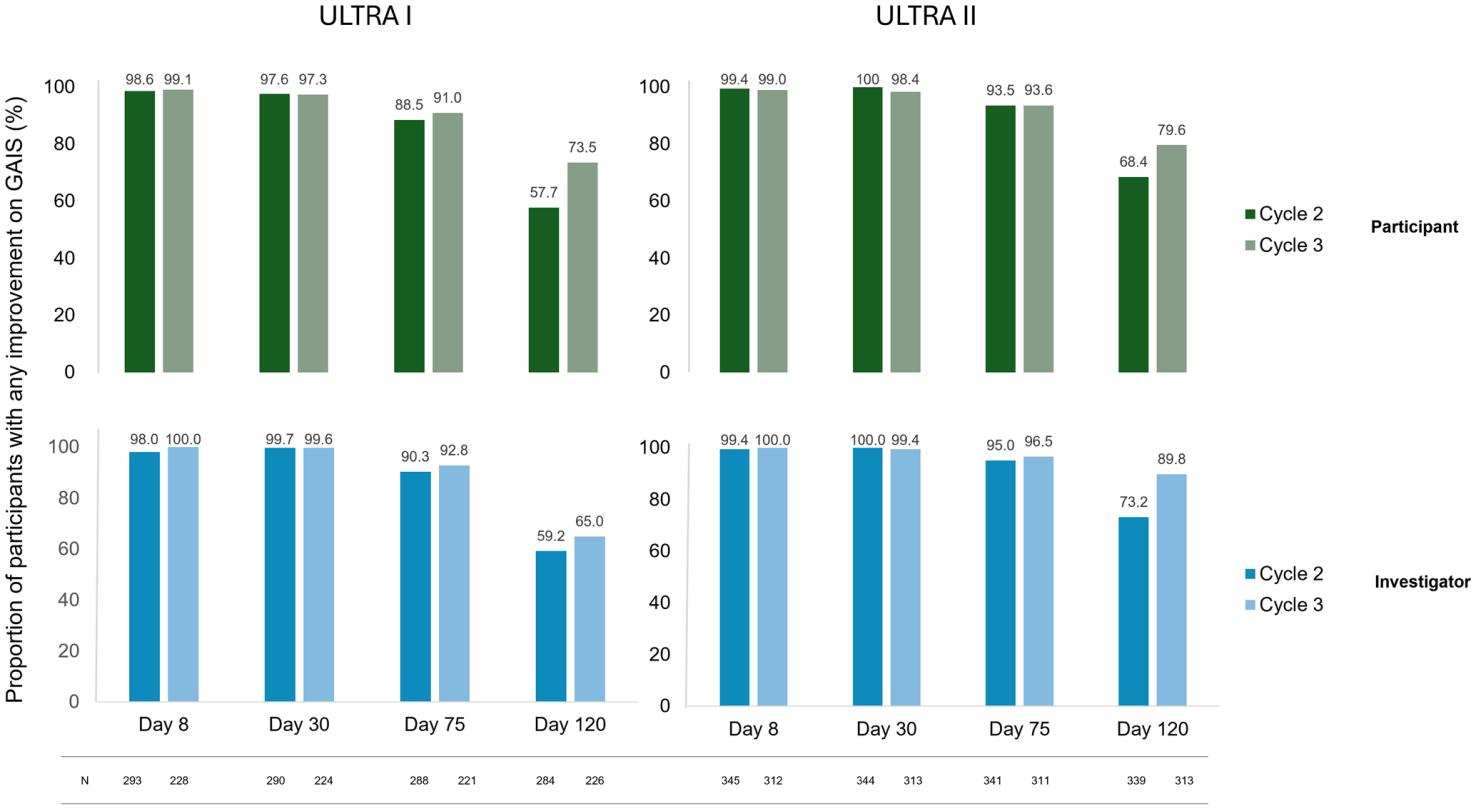
GAIS, “any improvement”^a^ over ULTRA I/II OLEX. This figure presents the rate of participants with “any improvement”^a^ on the GAIS in the OLEXs of ULTRA I/II, as assessed by the participant (above, green) and investigator (below, blue). As treatment was open‐label, participants were pooled into one treatment group. Analysis is based on observed cases in the full analysis set. ^a^Score of at least +1. GAIS, Global Aesthetic Improvement Scale; *N*, number of participants in respective analysis set; OLEX, open‐label extension period.

In the OLEX of both trials, the majority of participants (> 88%) showed any improvement in pGAIS and iGAIS to Day 75 of each cycle (Figure 3). Cycle 2 and Cycle 3 results were comparable.

**FIGURE 4:**
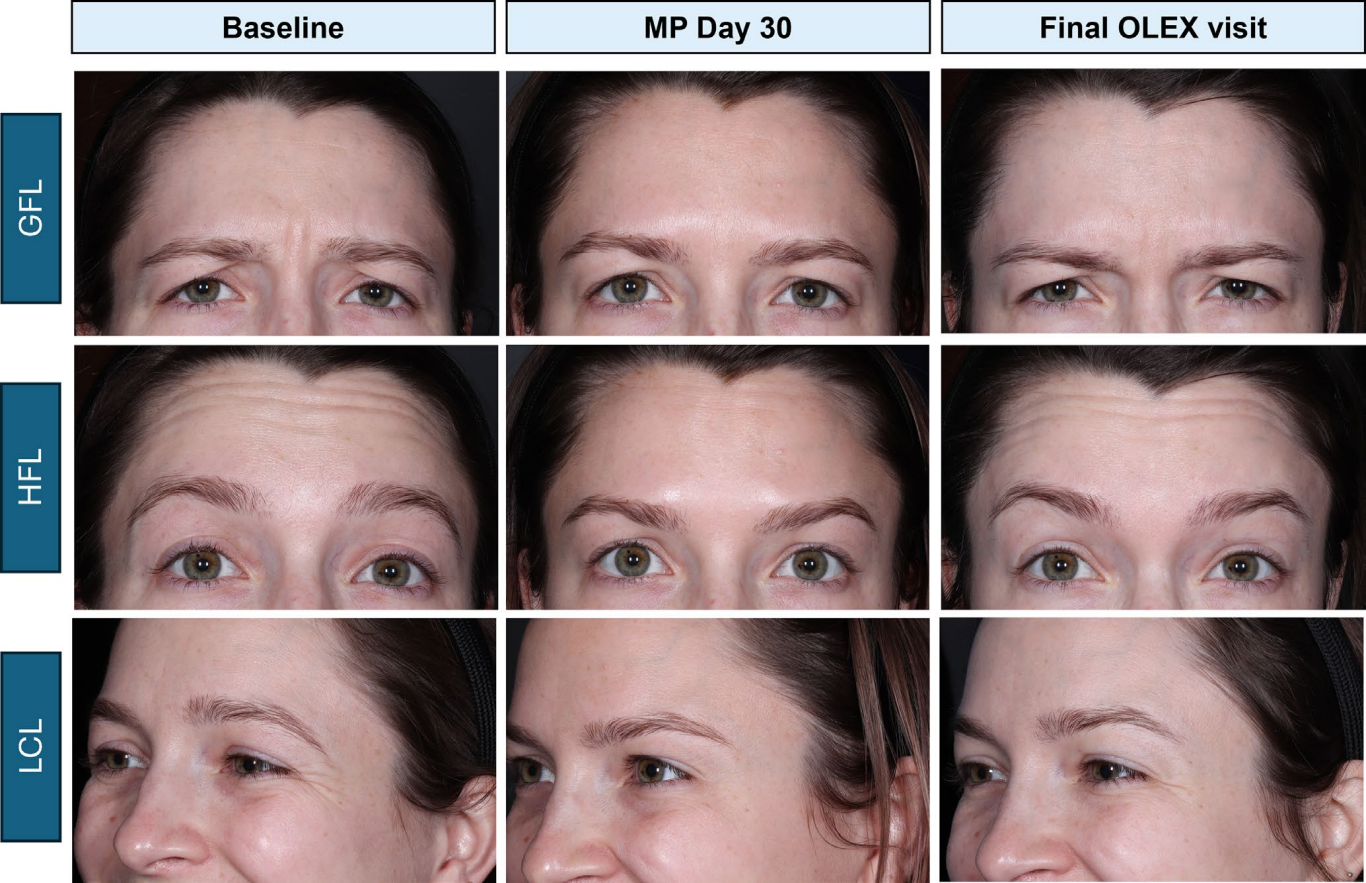
Representative photographs of one participant from Group U at maximum contraction at baseline, Day 30 of the MP and final OLEX visit (Cycle 3, Day 120). This figure shows photographs of a participant initially randomized to Group U, at maximum contraction for each respective treatment area (GFLs, HFLs and LCLs). Photos are from baseline, Day 30 of the MP and the final OLEX visit, following a total of 3 treatment cycles. GFL, glabellar frown lines; HFL, horizontal forehead lines; LCL, lateral canthal lines; MP, main period; OLEX, open‐label extension.

Representative participant photographs at maximum contraction, used for GAIS assessments at baseline and Day 30 of the MP and the OLEX final visit (Cycle 3, Day 120) are shown in Figure 4 and Figure S1.

### 3.3 MAS Endpoints

#### 3.3.1 Investigator Assessment at Maximum Contraction

For both trials, the rates of participants with a positive treatment response (at least a 1‐grade improvement in MAS from baseline) in the MP for GFLs, HFLs, and LCLs (combined for both left and right LCLs) at maximum contraction, as assessed by the investigator, are presented in Table 2.

**TABLE 2:** GFL, HFL and LCL improvement on MAS^a^ from baseline (investigator assessment at maximum contraction).

Facial area	Day	ULTRA I				ULTRA II			
		Main period							
		Treatment	*n*/*n*‐obs	%	95% CI^b^	Treatment	*n*/*n*‐obs	%	95% CI^b^
GFL	Day 8	Group P^c^ (*N* = 91)	3/88	3.4	1.2, 9.5	Group P^c^ (*N* = 94)	4/94	4.3	1.7, 10.4
	Day 30		1/89	1.1	0.2, 6.1		5/91	5.5	2.4, 12.2
	Day 60		4/86	4.7	1.8, 11.4		11/91	12.1	6.9, 20.4
	Day 90		2/87	2.3	0.6, 8.0		11/91	12.1	6.9, 20.4
	Day 120		5/88	5.7	2.5, 12.6		7/90	7.8	3.8, 15.2
	Day 8	Group U^c^ (*N* = 179)	166/175	94.9	90.5, 97.3	Group U^c^ (*N* = 184)	170/182	93.4	88.8, 96.2
	Day 30		167/173	96.5	92.6, 98.4		175/181	96.7	93.0, 98.5
	Day 60		161/173	93.1	88.3, 96.0		169/179	94.4	90.0, 96.9
	Day 90		136/170	80.0	73.4, 85.3		149/180	82.8	76.6, 87.6
	Day 120		90/170	52.9	45.5, 60.3		88/180	48.9	41.7, 56.1
HFL	Day 8	Group P^c^ (*N* = 91)	3/88	3.4	1.2, 9.5	Group P^c^ (*N* = 94)	3/94	3.2	1.1, 9.0
	Day 30		2/89	2.2	0.6, 7.8		3/91	3.3	1.1, 9.2
	Day 60		5/86	5.8	2.5, 12.9		6/91	6.6	3.1, 13.6
	Day 90		3/87	3.4	1.2, 9.7		8/91	8.8	4.5, 16.4
	Day 120		5/88	5.7	2.5, 12.6		2/90	2.2	0.6, 7.7
	Day 8	Group U^c^ (*N* = 179)	169/175	96.6	92.7, 98.4	Group U^c^ (*N* = 184)	176/182	96.7	93.0, 98.5
	Day 30		166/173	96.0	91.9, 98.0		174/181	96.1	92.2, 98.1
	Day 60		161/173	93.1	88.3, 96.0		164/179	91.6	86.6, 94.9
	Day 90		137/170	80.6	74.0, 85.8		141/180	78.3	71.8, 83.7
	Day 120		117/170	68.8	61.5, 75.3		86/180	47.8	40.6, 55.0
LCL	Day 8	Group P^c^ (*N* = 91)	8/88	9.1	4.7, 16.9	Group P^c^ (*N* = 94)	6/94	6.4	3.0, 13.2
	Day 30		7/89	7.9	3.9, 15.4		8/91	8.8	4.5, 16.4
	Day 60		10/86	11.6	6.4, 20.1		6/91	6.6	3.1, 13.6
	Day 90		11/87	12.6	7.2, 21.2		6/91	6.6	3.1, 13.6
	Day 120		11/88	12.5	7.1, 21.0		7/90	7.8	3.8, 15.2
	Day 8	Group U^c^ (*N* = 179)	151/175	86.3	80.4, 90.6	Group U^c^ (*N* = 184)	160/182	87.9	82.4, 91.9
	Day 30		154/173	89.0	83.5, 92.9		166/181	91.7	86.8, 94.9
	Day 60		134/173	77.5	70.7, 83.0		154/179	86.0	80.2, 90.4
	Day 90		108/170	63.5	56.1, 70.4		119/180	66.1	58.9, 72.6
	Day 120		67/170	39.4	32.4, 46.9		57/180	31.7	25.3, 38.8
Open‐label extension period									
GFL	Cycle 2 Day 30	Total Inco (*N* = 303)	283/290	97.6	95.1, 98.8	Total Inco (*N* = 346)	337/344	98.0	95.9, 99.0
	Cycle 3 Day 30	Total Inco (*N* = 238)	214/224	95.5	92.0, 97.6	Total Inco (*N* = 318)	306/313	97.8	95.5, 98.9
HFL	Cycle 2 Day 30	Total Inco (*N* = 303)	279/290	96.2	93.3, 97.9	Total Inco (*N* = 346)	340/344	98.8	97.0, 99.5
	Cycle 3 Day 30	Total Inco (*N* = 238)	219/224	97.8	94.9, 99.0	Total Inco (*N* = 318)	300/313	95.8	93.0, 97.6
LCL	Cycle 2 Day 30	Total Inco (*N* = 303)	276/290	95.2	92.1, 97.1	Total Inco (*N* = 346)	331/344	96.2	93.6, 97.8
	Cycle 3 Day 30	Total Inco (*N* = 238)	206/224	92.0	87.7, 94.9	Total Inco (*N* = 238)	293/313	93.6	90.3, 95.8

*Note:* This table presents the rate of participants with “improvement”^a^ on MAS, as assessed by the investigator, over the MP and OLEX cycles. Participants were assessed at maximum contraction, i.e., participants were required to tense the treated facial area. As treatment was open‐label in the OLEX, participants were pooled into one treatment group, which was renamed Total Inco. Analysis is based on observed cases in the full analysis set.

Abbreviations: CI, confidence interval; GFL, glabellar frown lines; HFL, horizontal forehead lines; inco, incobotulinumtoxinA; LCL, lateral canthal lines; MAS, Merz Aesthetics Scale; MP, main period; *N*, number of participants in respective analysis set, and for OLEX period those treated in respective cycle; *n*, number of responders; *n*‐obs, number of observed cases; OLEX, open‐label extension.

^a^ At least 1‐grade.

^b^ Wilson CI.

^c^ Group P: placebo group, Group U: upper facial lines (a combination of glabellar frown lines and horizontal forehead lines and lateral canthal lines) treated.

For ULTRA I MP, the majority of participants in Group U had a positive treatment response for GFLs, HFLs, and LCLs up to Day 120. For GFLs and HFLs, > 90% of participants in Group U showed a response at Days 8, 30, and 60. At Day 90, rates remained high and > 80% of participants had a positive response for GFLs and HFLs. At Day 120, the rates of participants with a response declined (52.9% for GFLs and 68.8% for HFLs). The rate of participants with a positive response for GFLs and HFLs in Group P remained low at all time points (< 6%). For LCLs, > 77% of participants in Group U had a response at Days 8, 30, and 60, which decreased to 63.5% at Day 90 and to 39.4% at Day 120. The rate of participants in Group P with a response for LCLs was ~10% at all time points.

For ULTRA II MP, similar results were observed, with the majority of treated participants experiencing a positive treatment response for GFLs, HFLs, and LCLs to Day 120 (Table 2). For GFLs and HFLs, > 90% of participants in Group U showed a response at Days 8, 30, and 60, and > 78% at Day 90. At Day 120, the rate of participants with a response declined to 47.8% for HFLs and 48.9% for GFLs. The rates of participants in Group P with a response for GFLs and HFLs remained low at all timepoints (< 13%). For LCLs, > 86% of participants in Group U showed a response at Days 8, 30, and 60, which decreased to 66.1% at Day 90 and to 31.7% at Day 120. The rate of participants in Group P with improvement in LCLs ranged from 6.6% to 8.8%.

For both OLEX periods, results across Cycles 2 and 3 were consistent with those seen for Group U in the MP (Table 2). At Day 30 for Total Inco, rates of participants with a positive treatment response were high (> 92%).

#### 3.3.2 Participant Assessment at Maximum Contraction

For both trials, the rates of participants with a positive treatment response for GFLs, HFLs, and LCLs (combined for both left and right LCLs) at maximum contraction, as assessed by the participant, are presented in Table S1 for the MP and OLEX. Participant‐assessed MAS showed a similar pattern to the investigator assessment.

## 4 Discussion

The current results demonstrate aesthetic improvement of UFLs after simultaneous treatment with incobotulinumtoxinA, as reported by the participant and the investigator. These data add to the pivotal data published previously [10], providing further information on the sustained treatment effects and participant‐assessed endpoints.

Satisfaction with aesthetic outcome is an important indicator of treatment success. In aesthetic clinical trials, this success can be demonstrated by a ≥ 1‐grade improvement versus baseline using validated scales. While not assessed in ULTRA I and II, participants with treatment success may also experience improvements in psychosocial measures such as attractiveness and confidence, which contribute to overall improvements in wellbeing and quality of life (QoL) [13, 14]. A recent evidence‐based review of 11 studies showed that BoNT‐A treatment for facial aesthetic improvement resulted in numerical and/or statistically significant improvements in psychological wellbeing and QoL, with the most significant impact seen when multiple areas were treated in a multimodal approach [15].

In the current studies, the overall aesthetic improvement from the participant's and the investigator's perspective was evaluated by GAIS. This assessment captures the subjective experience of the participant, reflecting their personal satisfaction with the treatment outcomes. The GAIS score is used to holistically assess the aesthetic improvement of the whole face over time. The MPs of ULTRA I and II showed high rates of participants with an improvement in GAIS scores, reflecting an overall high satisfaction with the treatment outcome. However, GAIS scores were lower in Group L compared with Group G&H. This finding could be because only one region (LCLs) was treated in Group L versus two regions (GFLs and HFLs) in Group G&H, the latter of which reported higher participant satisfaction, ultimately reflected by higher pGAIS scores. The OLEX cycles also showed high rates of participants with improvement in GAIS scores (> 97% for both pGAIS and iGAIS at Day 30), indicating that simultaneous UFL treatment results in sustained aesthetic improvement over three cycles of treatment. From a practical perspective, this simultaneous treatment approach has a real‐world benefit, as fewer clinic visits are required, resulting in improved efficiency for the patient and the provider.

Participant‐assessed GAIS results also show that the treatment effect of 64 U incobotulinumtoxinA for the UFL indication (simultaneous treatment of the GFLs, HFLs and LCLs) was sustained over the three treatment cycles. These results indicate no signs of tachyphylaxis and demonstrate that the higher overall dose did not compromise aesthetic improvement (efficacy) or safety [10]. This data adds to the growing body of literature demonstrating that repeated botulinum toxin treatments are efficacious and well tolerated, which will aid clinicians and injectors in developing long‐term treatment plans to attain optimal aesthetic outcomes.

Results were largely similar between the pGAIS and iGAIS, indicating that both the participant and the investigator were able to discern posttreatment aesthetic improvement. However, pGAIS scores were slightly lower than iGAIS scores. Participants generally have a more critical bias toward the severity of their treatment concerns, a finding that has been observed in other studies of incobotulinumtoxinA, daxibotulinumtoxinA, and abobotulinumtoxinA [16, 17, 18]. Investigators, trained to apply photonumeric scales objectively, are less likely to be influenced by personal self‐criticism or emotional self‐discrepancy. In contrast, participants often compare themselves against an internalized “ideal” image, leading to more conservative or critical personal ratings [19]. Looking ahead, using artificial intelligence to comprehensively analyze facial features could yield a more objective aesthetic evaluation [20, 21].

Minor differences in GAIS scores were observed over the MP between the ULTRA I and II, which could be attributed to cultural differences and a tendency for North Americans to offer more positive self‐assessments [22]. This bias appears less pronounced in European countries, including Germany. Additionally, studies show that cultural norms around body image and cosmetic procedures vary [23]; these norms could potentially influence how people evaluate aesthetic improvements.

Investigator‐assessed MAS results at maximum contraction showed most treated participants had a positive response in the MP for each treatment area (HFLs, GFLs, and LCLs) to Day 120. Results from both trials identified that responses were generally similar for HFLs and GFLs, with slightly lower responses observed for the LCLs. As the 24 U for the LCL area was halved (12 U per side), the LCLs received a relatively low dose when compared to the 20 U received in the HFLs and GFLs. Although this dose is in line with recommended BoNT‐A dosing for LCLs to ensure that eyelid movement remains unaffected, the smaller dose may not have the same duration of effect as the higher doses used in the other areas [24, 25]. BoNT‐A dosages vary across treatment indications due to the intricate facial musculature, and onset can differ in the various facial areas [2, 26, 27, 28]. The differences in the facial musculature are extensive; GFLs are short, thick muscles known as the procerus and corrugators, HFLs are governed by a large, thin, frontalis muscle, and LCLs by the sheet‐like orbicularis oculi [26, 27]. The muscles may also differ in contraction strength between and within individuals [27]. Therefore, anatomical and functional distinctions underscore the importance of tailored injection strategies and highlight the variability in treatment response across facial regions [29].

Using variable injection sites and depths to target relevant muscles, BoNT‐A treatment can be customized for the participant's age and muscle volume [2, 26, 27]. In a recent case series of 20 participants receiving incobotulinumtoxinA for simultaneous treatment of HFLs and GFLs, the highest percentages of MAS responders were observed at Day 15 for HFLs (90%) and Day 90 for GFLs (95%), suggesting that the onset of treatment effect can vary by region [30]. In contrast, another study assessing open‐label incobotulinumtoxinA for UFLs treatment showed very similar MAS responses in all three areas (96%, 100% and 100% for HFLs, GFLs and LCLs, respectively) at Day 30, with participant satisfaction reported through 180 days, which the authors attribute to the use of a personalized injection technique [31]. These studies suggest that although efficacy onset may differ by facial area, it can be mitigated in real‐world implementation.

Study limitations include the predominantly female (> 80% in both trials) participant demographic; although representative of the population seeking aesthetic procedures, future studies could aim to recruit more male participants. Anatomic differences, such as increased muscle mass, duration of treatment effect, and onset of effect, are a few factors to consider when treating males for aesthetic indications [32, 33, 34].

## 5 Conclusion

Additional endpoints from the two Phase III ULTRA trials support the initial conclusions of safe and effective simultaneous BoNT‐A treatment for UFLs, with high participant ratings of aesthetic improvement and high agreement between investigator‐ and participant‐assessed outcomes over time. Additionally, the BoNT‐A treatment effect was shown to be maintained over the OLEX cycles.

## Author Contributions

T.P. contributed to conception or design of the work. E.K.B., T.P., M.S.N., H.D. and V.S. contributed to the acquisition, analysis, or interpretation of data for the work. All authors drafted and reviewed the work critically for important intellectual content; and all authors provided final approval of the version to be published and agreed to be accountable for all aspects of the work in ensuring that questions related to the accuracy or integrity of any part of the work are appropriately investigated and resolved.

## Ethics Statement

The protocols were approved by the institutional review boards and/or independent ethics committees: ULTRA I, Advarra, number Pro00044813 (ethics approval received 02 July 2020); and ULTRA II, the Ethics Committee of the Hamburg Medical Association, number PVN7390 (ethics approval received 17 August 2020). All participants provided written informed consent, and the study was conducted in compliance with Good Clinical Practice and the Declaration of Helsinki. Participant consent was obtained for the use of photographs included in the publication.

## Conflicts of Interest

T.P. is a consultant and lecturer for Merz Aesthetics GmbH. C.B. is a Merz Aesthetics Principal Investigator. S.F. is a consultant, lecturer and investigator for Merz Aesthetics GmbH. M.S.N. has received research grants from Merz Aesthetics GmbH. E.K.B. is a consultant for Merz Aesthetics GmbH. M.I. is a speaker for Merz Aesthetics GmbH. V.S. and H.D. are employees of Merz Aesthetics GmbH.

## Supporting information


**Figure S1:** Representative participant photographs of three individual participants at maximum contraction at Baseline, Day 30 of the MP and final OLEX visit (Cycle 3, Day 120). This figure shows representative participant photographs from ULTRA I/II at maximum contraction for each respective treatment area. Photos are from baseline, Day 30 of the MP and the final OLEX visit, following a total of three treatment cycles.
**Table S1:** GFL, HFL, and LCL on MAS^a^ from baseline over MP (participant assessment at maximum contraction).

## Data Availability

Data supporting this study will not be made available publicly. Data may be made available to authorities upon request. These requests are reviewed and approved by an independent review panel on the basis of scientific merit. All data provided are anonymized to respect the privacy of participants who took part in the trial, in line with applicable laws and regulations. This trial data availability is according to the criteria and process described on www.clinicalstudydatarequest.com.
