# Supplementary material for: Aesthetic Improvements Over Time: Long‐Term Efficacy and Additional Outcomes of IncobotulinumtoxinA in the Simultaneous Treatment of Upper Facial Lines
Source: J Cosmet Dermatol. 2025 Sep 18;24(9):e70460. doi: 10.1111/jocd.70460 (PMC12446747; doi:10.1111/jocd.70460)
Supplement: Supplementary file 1 — Figure S1: Representative participant photographs of three individual participants at maximum contraction at Baseline, Day 30 of the MP and final OLEX visit (Cycle 3, Day 120). This figure shows representative participant photographs from ULTRA I/II at maximum contraction for each respective treatment area. Photos are from baseline, Day 30 of the MP and the final OLEX visit, following a total of three treatment cycles. Table S1: GFL, HFL, and LCL on MASa from baseline over MP (participant assessment at maximum contraction). [file JOCD-24-e70460-s001.docx]

**Supplementary**

**Supplementary Table 1. GFL, HFL and LCL on MAS^a^ from baseline over MP and OLEX (participant assessment at maximum contraction)**

|  |  | **ULTRA I** | | | | **ULTRA II** | | | |
| --- | --- | --- | --- | --- | --- | --- | --- | --- | --- |
|  |  | **Main Period** | | | | | | | |
| **Facial area** | **Day** | **Treatment** | **n/n-obs** | **%** | **95%CI^b^** | **Treatment** | **n/n-obs** | **%** | **95%CI^b^** |
| GFL | Day 8 | Group P^c^  (N=91) | 9/88 | 10.2 | 5.5, 18.3 | Group P^c^  (N=94) | 9/94 | 9.6 | 5.1, 17.2 |
|  | Day 30 |  | 7/89 | 7.9 | 3.9, 15.4 |  | 6/92 | 6.5 | 3.0, 13.5 |
|  | Day 60 |  | 4/86 | 4.7 | 1.8, 11.4 |  | 9/91 | 9.9 | 5.3, 17.7 |
|  | Day 90 |  | 4/87 | 4.6 | 1.8, 11.2 |  | 8/91 | 8.8 | 4.5, 16.4 |
|  | Day 120 |  | 6/88 | 6.8 | 3.2, 14.1 |  | 8/90 | 8.9 | 4.6, 16.6 |
|  | Day 8 | Group U^c^ (N=179) | 156/175 | 89.1 | 83.7, 92.9 | Group U^c^ (N=184) | 156/182 | 85.7 | 79.9, 90.1 |
|  | Day 30 |  | 156/173 | 90.2 | 84.8, 93.8 |  | 170/181 | 93.9 | 89.4, 96.6 |
|  | Day 60 |  | 141/173 | 81.5 | 75.1, 86.6 |  | 154/179 | 86.0 | 80.2, 90.4 |
|  | Day 90 |  | 108/170 | 63.5 | 56.1, 70.4 |  | 138/180 | 76.7 | 70.0, 82.2 |
|  | Day 120 |  | 77/170 | 45.3 | 38.0, 52.8 |  | 74/180 | 41.1 | 34.2, 48.4 |
| HFL | Day 8 | Group P^c^  (N=91) | 10/88 | 11.4 | 6.3, 19.7 | Group P^c^  (N=94) | 7/94 | 7.4 | 3.7, 14.6 |
|  | Day 30 |  | 7/89 | 7.9 | 3.9, 15.4 |  | 12/94 | 13.0 | 7.6, 21.4 |
|  | Day 60 |  | 9/86 | 10.5 | 5.6, 18.7 |  | 14/91 | 15.4 | 9.4, 24.2 |
|  | Day 90 |  | 6/87 | 6.9 | 3.2, 14.2 |  | 15/91 | 16.5 | 10.3, 25.4 |
|  | Day 120 |  | 10/88 | 11.4 | 6.3, 19.7 |  | 11/90 | 12.2 | 7.0, 20.6 |
|  | Day 8 | Group U^c^ (N=179) | 163/175 | 93.1 | 88.4, 96.0 | Group U^c^ (N=184) | 165/182 | 90.7 | 85.6, 94.1 |
|  | Day 30 |  | 162/173 | 93.6 | 89.0, 96.4 |  | 171/181 | 94.5 | 90.1, 97.0 |
|  | Day 60 |  | 147/173 | 85.0 | 78.9, 89.5 |  | 162/179 | 90.5 | 85.3, 94.0 |
|  | Day 90 |  | 123/170 | 72.4 | 65.2, 78.5 |  | 134/180 | 74.4 | 67.6, 80.3 |
|  | Day 120 |  | 85/170 | 50.0 | 42.6, 57.4 |  | 78/180 | 43.3 | 36.3, 50.6 |
| LCL | Day 8 | Group P^c^  (N=91) | 11/88 | 12.5 | 7.1, 21.0 | Group P^c^ (N=94) | 9/94 | 9.6 | 5.1, 17.2 |
|  | Day 30 |  | 10/89 | 11.2 | 6.2, 19.5 |  | 10/92 | 10.9 | 6.0, 18.9 |
|  | Day 60 |  | 11/86 | 12.8 | 7.3, 21.5 |  | 7/91 | 7.7 | 3.8, 15.0 |
|  | Day 90 |  | 7/87 | 8.0 | 4.0, 15.7 |  | 8/91 | 8.8 | 4.5, 16.4 |
|  | Day 120 |  | 8/88 | 9.1 | 4.7, 16.9 |  | 9/90 | 10.0 | 5.4, 17.9 |
|  | Day 8 | Group U^c^ (N=179) | 137/175 | 78.3 | 71.6, 83.8 | Group U^c^ (N=184) | 138/182 | 75.8 | 69.1, 81.5 |
|  | Day 30 |  | 139/173 | 80.8 | 74.3, 86.0 |  | 158/181 | 87.3 | 81.7, 91.4 |
|  | Day 60 |  | 129/173 | 74.6 | 67.6, 80.5 |  | 138/179 | 77.1 | 70.4, 82.6 |
|  | Day 90 |  | 100/170 | 58.8 | 51.3, 65.9 |  | 108/180 | 60.0 | 52.7, 66.9 |
|  | Day 120 |  | 69/170 | 40.6 | 33.5, 48.1 |  | 58/180 | 32.2 | 25.8, 39.4 |
|  |  | **Open-Label Extension Period** | | | | | | | |
| GFL | Cycle 2 Day 30 | Total Inco  (N=303) | 272/290 | 93.8 | 90.4, 96.0 | Total Inco  (N=346) | 328/344 | 95.3 | 92.6, 97.1 |
|  | Cycle 3 Day 30 | Total Inco  (N=238) | 198/224 | 88.4 | 83.5, 92.0 | Total Inco  (N=318) | 293/313 | 93.6 | 90.3, 95.8 |
| HFL | Cycle 2 Day 30 | Total Inco  (N=303) | 270/290 | 93.1 | 89.6, 95.5 | Total Inco  (N=346) | 334/344 | 97.1 | 94.7, 98.4 |
|  | Cycle 3 Day 30 | Total Inco  (N=238) | 203/224 | 90.6 | 86.1, 93.8 | Total Inco  (N=318) | 284/313 | 90.7 | 87.0, 93.5 |
| LCL | Cycle 2 Day 30 | Total Inco  (N=303) | 252/290 | 86.9 | 82.5, 90.3 | Total Inco  (N=346) | 315/344 | 91.6 | 88.2, 94.1 |
|  | Cycle 3 Day 30 | Total Inco  (N=238) | 185/224 | 82.6 | 77.1, 87.0 | Total Inco  (N=238) | 274/313 | 87.5 | 83.4, 90.7 |

Supplementary Table 1 presents the rate of participants with “improvement”^a^ on MAS, as assessed by the participant, over the MP and OLEX cycles. Participants assessed themselves at maximum contraction, i.e. participants were required to tense the treated facial area. As treatment was open-label in the OLEX, participants were pooled into one treatment group, which was renamed Total Inco. Analysis is based on observed cases in the full analysis set.

^a^At least 1-grade improvement.

^b^Wilson CI.

^c^Group P: placebo group, Group U: upper facial lines (a combination of glabellar frown lines and horizontal forehead lines and lateral canthal lines) treated.

Abbreviations: CI, confidence interval; GFL, glabellar frown lines; HFL, horizontal forehead lines; LCL, lateral canthal lines; MAS, Merz Aesthetics Scale; MP; main period; n, number of responders; N, number of participants in respective analysis set, and for OLEX period those treated in respective cycle; n-obs, number of observed cases.

**Supplementary Figure 1. Representative participant photographs of three individual participants at maximum contraction at Baseline, Day 30 of the MP and final OLEX visit (Cycle 3, Day 120)**


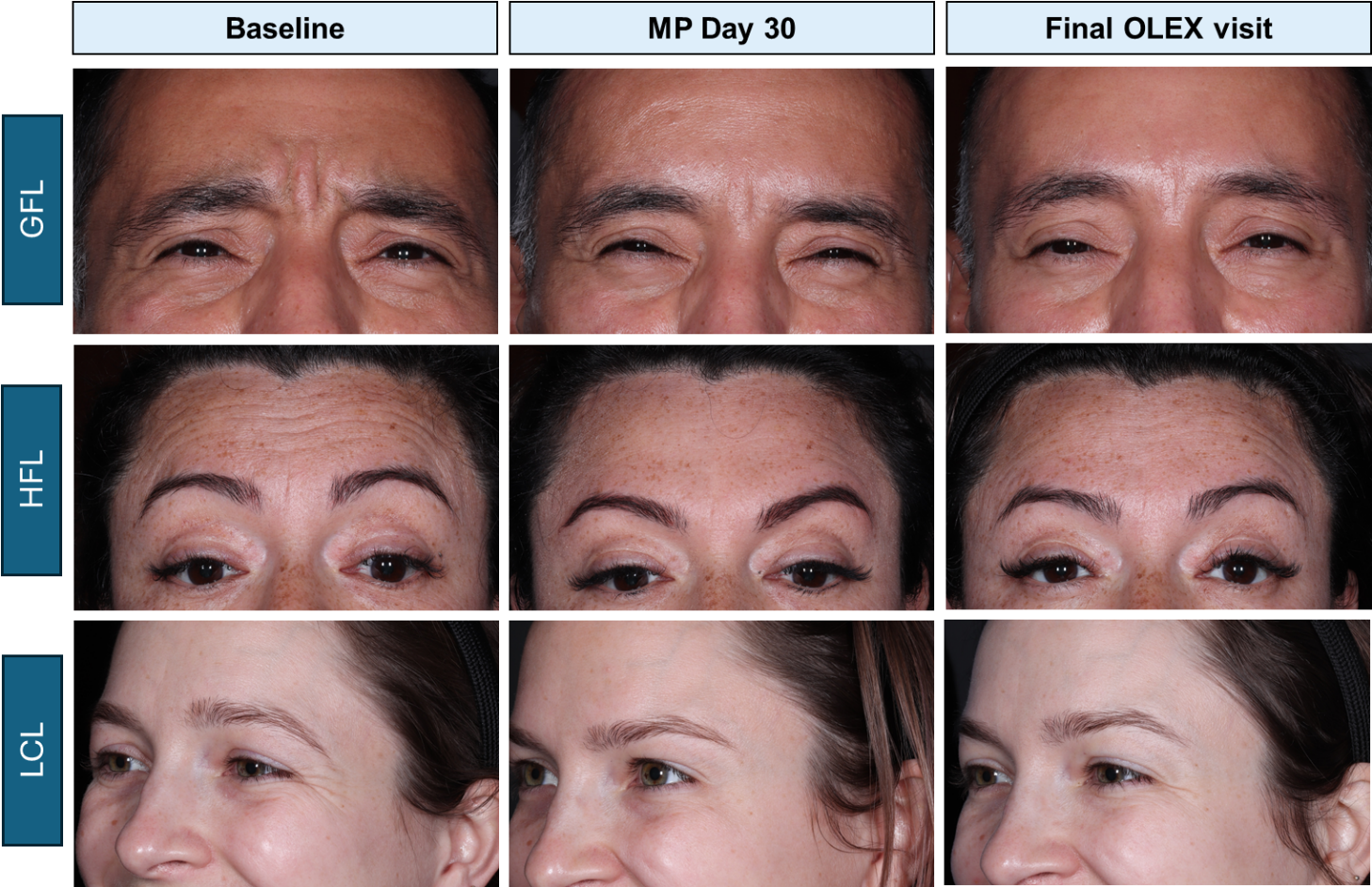


Supplementary Figure 1 shows representative participant photographs from ULTRA I/II at maximum contraction for each respective treatment area. Photos are from Baseline, Day 30 of the MP and the final OLEX visit, following a total of 3 treatment cycles.

Abbreviations: GFL, glabellar frown lines; HFL, horizontal forehead lines; LCL, lateral canthal lines; MP, main period; OLEX, open-label extension.
